# Inconsistency of Karyotyping and Array Comparative Genomic Hybridization (aCGH) in a Mosaic Turner Syndrome Case

**DOI:** 10.1055/s-0041-1722974

**Published:** 2021-02-11

**Authors:** Pinar Tulay, Mahmut Cerkez Ergoren, Ahmet Alkaya, Eyup Yayci, Sebnem Ozemri Sag, Sehime Gulsum Temel

**Affiliations:** 1 Near East University, Faculty of Medicine, Department of Medical Genetics, Nicosia, Cyprus; 2 Near East University, DESAM Institute, Nicosia, Cyprus; 3 Bilecik Seyh Edebali University, Graduate School of Applied Sciences, Gulumbe Yerleskesi, Bilecik, Turkey; 4 Near East University, Faculty of Medicine, Department of Gynecology and Obstetrics, Nicosia, Cyprus; 5 Uludag University, Faculty of Medicine, Department of Medical Genetics, Bursa, Turkey; 6 Uludag University, Faculty of Medicine, Department of Histology and Embryology, Bursa, Turkey

**Keywords:** turner syndrome, mosaicism, partial mosaicism, aCGH, cytogenetics

## Abstract

**Purpose** Turner syndrome is a sex chromosomal aberration where majority of the patients have 45,X karyotype, while several patients are mosaic involving 45,X/46,XX; 46,X,i(Xq); and other variants. Cytogenetic analysis, karyotyping, is considered to be the “gold standard” to detect numerical and structural chromosomal abnormalities. In the recent years, alternative approaches, such as array comparative genomic hybridization (aCGH), have been widely used in genetic analysis to detect numerical abnormalities as well as unbalanced structural rearrangements. In this study, we report the use of karyotyping as well as aCGH in detecting a possible Turner syndrome variant.

**Methods** An apparent 16-year-old female was clinically diagnosed as Turner syndrome with premature ovarian failure and short stature. The genetic diagnosis was performed for the patient and the parents by karyotyping analysis. aCGH was also performed for the patient.

**Main Findings** Cytogenetic analysis of the patient was performed showing variant Turner syndrome (46,X,i(X)(q10)[26]/46,X,del(X)(q11.2)[11]/45,X[8]/46,XX[5]). The patient's aCGH result revealed that she has a deletion of 57,252kb of Xp22.33-p11.21 region; arr[GRCh37] Xp22.33-p11.21 (310,932–57,563–078)X1. Both aCGH and fluorescence in situ hybridization (FISH) results suggested that *short stature Homeobox-containing* ( *SHOX* ) gene, which is located on Xp22.33, was deleted, though FISH result indicated that this was in a mosaic pattern.

**Conclusion** In the recent years, aCGH has become the preferred method in detecting numerical abnormalities and unbalanced chromosomal rearrangements. However, its use is hindered by its failure of detecting mosaicism, especially low-level partial mosaicism. Therefore, although the resolution of the aCGH is higher, the cytogenetic investigation is still the first in line to detect mosaicism.

## Introduction

Turner syndrome is the most common sex chromosome condition in females with a prevalence of 1 in 2,500 live births. 1 Turner syndrome patients have distinct phenotype of webbed neck, low posterior hairline, and broad chest. The haploinsufficiency of *SHOX* ( *short stature Homeobox-containing* gene) is directly associated with the short stature of Turner syndrome patients. In addition to skeletal anomalies, *SHOX* haploinsufficiency is also associated with hearing impairment. 2 Abnormalities of secondary sex characteristics, structural cardiac anomalies, hypertension, diabetes, renal anomalies, and hearing loss are also observed in Turner syndrome patients. 3 Due to the multidisciplinary medical problems, during childhood, the girls are routinely screened. 4 However, the screening seems to fail during adulthood with only as few as 4% females being screened as recommended yearly. 3 5

The routine genetic diagnosis of Turner syndrome is performed by conventional cytogenetic analysis. In the last decade, alternative approaches, including polymerase chain reaction-based techniques and single nucleotide polymorphism (SNP) genotyping, have been developed to detect sex chromosome abnormalities in a timely and cost-effective way using both blood and buccal samples. With the wider use of microarray analysis, the array comparative genomic hybridization (aCGH) has been used to diagnose the Turner syndrome patients genetically. aCGH has advantages over conventional karyotyping analysis since it is less labor intensive with the ability to detect deletions and duplications that are less than 1 to 2Mb. 6 Although the resolution of aCGH is better compared with conventional karyotype analysis, the detection of mosaic chromosome complement has proven to be more difficult using aCGH. Mosaic Turner Syndrome cases arise as a random event during cleavage stage divisions in early embryogenesis resulting in patients with a mosaic karyotype of 45,X/46,XX, 46,X,i(Xq) and other variants. 7 Overall, the mosaic Turner Syndrome patients may be under-diagnosed not only due to the subtle phenotypic characteristics but also due to technical limitations. 8 This becomes more apparent in patients with low rates of mosaicism since high number of euploid cells may mask the true genetic diagnosis or it may be reported as an artifact. To date, several studies have reported that aCGH could detect mosaicism with varying rates depending on the array platform, such as 75% ring chromosome 18q, 9 72% marker chromosome with chromosome 17p origin, 10 30% trisomy 20, 11 21% rearranged chromosome 18 12 and even 7% monosomy 7. 13 In this study, a patient with a suspected Turner syndrome variant was genetically diagnosed by both conventional cytogenetic and aCGH analyses.

## Case Presentation

A 16-year-old apparent female patient with a clinical Turner syndrome phenotype was referred for genetic counselling. The clinical evaluation revealed that the patient was 147 cm in height. She was diagnosed with premature ovarian failure and she was treated with hormonal replacement therapy followed by menstruation at the age of 14 years. Her follicle-stimulating hormone levels were reported to be 155 IU/L, luteinizing hormone was 23.34 IU/L, and estradiol was 11pg/mL, respectively. She was diagnosed with arrhythmia and ablation by cardiologist.

Whole blood was obtained from the patient, the mother, and the father for cytogenetics analysis following getting consent forms. The lymphocytes were cultured and metaphase chromosomes were obtained by phytohaemagglutinin (PHA) stimulation. Standard GTL banding was performed at 400 to 500 band level. Fluorescence in situ hybridization analysis was performed for the patient on both metaphase and interphase chromosome spreads using probes specific for; *SHOX* gene located on Xp22.33/Yp11.32, DYZ1 on Yq12, and DXZ1 on Xp11.2-q11.1 according to manufacturer's protocol (CytoCell).

To compare the karyotype and aCGH results, additional blood sample was also collected from the patient. DNA sample was obtained (DNeasy blood and tissue kit) and the microarray analysis was performed according to manufacturer's protocol (Agilent Oligonucleotide Array-Based CGH for Genomic Analysis). The sample and the control DNA were labeled by ULS-Cy3 and ULS-Cy5 according to manufacturer's protocol. Agilent oligonucleotide microarray was used for the hybridization with 8*60K coverage for 24 hours at 65°C. Following microarray washes, the slides were scanned and analysis was performed using the Agilent CytoGenomic edition 5.0.0.38 analysis program.

**Fig. 1:**
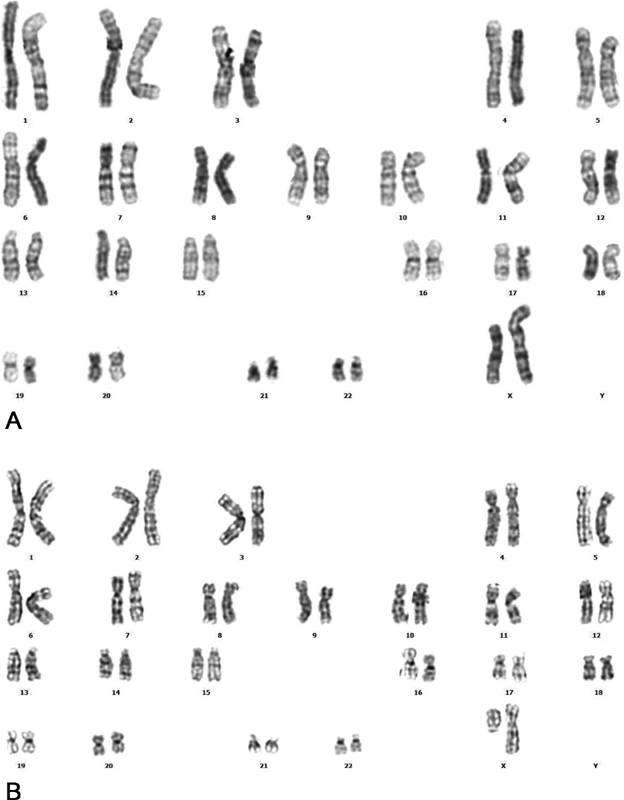
Karyotyping results of peripheral blood lymphocytes. The patient was shown to have 46,X,i(X)(q10)[26]/46,X,del(X)(q11.2)[11]/45,X[8]/46,XX[5]. ( **A** ) Karyotype analysis showing 46,X,del(X)(q11.2). ( **B** ) Karyotype analysis showing 46,X,i(X)(q10).

**Fig. 2:**
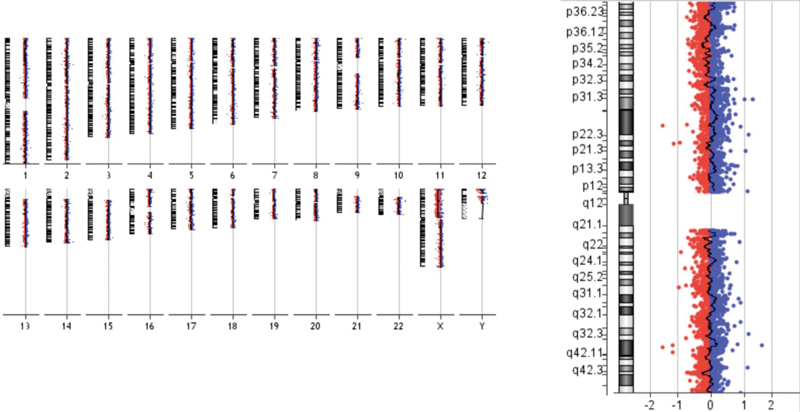
Array comparative genomic hybridization results showing 57,252kb of Xp22.33-p11.21 region was rather identified; arr[GRCh37] Xp22.33-p11.21 (310,932–57,563–078)X1.

**Fig. 3:**
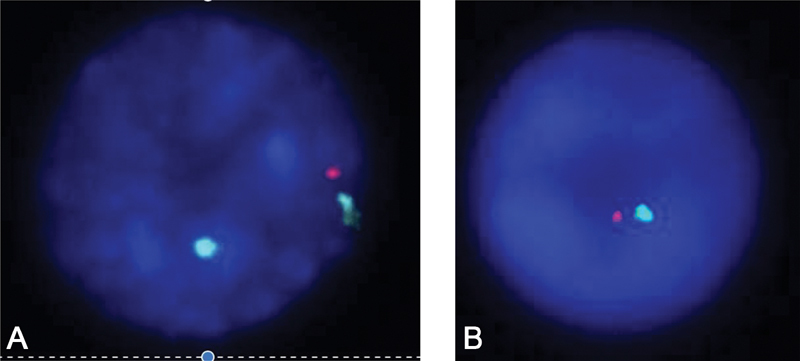
Fluorescence in situ hybridization (FISH) results using probes (CytoCell) of *short stature Homeobox-containing* (SHOX), Xp22.33/Yp11.32 (red), DXZ1, Xp11.1-q11.1 (blue) and DYZ1, Yq12 (green). ( **A** ) FISH result showing deletion of one copy of *SHOX* gene on metaphase chromosome spread. ( **B** ) FISH result showing monosomy X on interphase chromosome spread.

The 16-year-old patient was reported to have a mosaic karyotype with 52% (26/50) isochromosome Xq, 22% (11/50) terminal deletion of Xq11.2 region, 15% (8/50) monosomy X, and 10% (5/50) euploid karyotype; 46,X,i(X)(q10)[26]/46,X,del(X)(q11.2)[11]/45,X[8]/46,XX[5] ( Fig. 1 ). The karyotyping result of this patient was in agreement with variant Turner syndrome. However, the aCGH analysis did not detect any mosaicism. A deletion of 57,252kb of Xp22.33-p11.21 region was rather identified; arr[GRCh37] Xp22.33-p11.21 (310,932–57,563–078)X1 ( Fig. 2 ). A total of 3,370 probes were present in this region. Therefore, aCGH result suggested that *SHOX* gene, which is located on Xp22.33, was deleted. Fluorescence in situ hybridization analysis also confirmed the aCGH result of *SHOX* gene deletion, though in a mosaic pattern ( Fig. 3 ). The mother of the patient was shown to have a euploid karyotype; 46,XX; whereas the patient's father was reported to have an inversion on chromosome 2; 46,XY,inv(2)(p11.2q13).

## Discussion and Conclusions

Mosaicism has been challenging to identify since by definition some cells have the abnormality whereas the others do not. It is inherently difficult to detect the true levels of mosaicism since it can be confined to certain tissues. With the developing technologies, genomic analyses have been advanced to detect mosaicism, even at low levels. In this study, we present the genetic diagnosis of a patient with a Turner syndrome phenotype. Both conventional cytogenetic analysis and chromosomal microarray analysis were performed to diagnose this patient.

Conventional cytogenetic investigations are the gold standard to detect any chromosomal abnormalities, involving both structural and numerical. However, there has been a long debate on the use of cytogenetic techniques in detecting mosaicism. One of the main drawbacks of conventional cytogenetic analysis is that it depends on PHA-stimulated T cells. Therefore, there is a risk of bias following stimulation and culturing these cells leading to a possible misdiagnosis. 14 15 Furthermore, low level mosaicism may be missed by conventional cytogenetic analysis by visual inspection, especially with small marker chromosomes. Studies report that more number of cells are required to maximize the detection rate, in such at least 29 metaphase chromosomes have to be analyzed to detect 10% and 63 metaphase chromosomes for 5% mosaicism have to be analyzed with 95% confidence limit, respectively. 8 16 Due to these drawbacks, well-trained and experienced cytogeneticists are required for correct diagnosis. All these makes the cytogenetics analysis labor-intensive, time consuming, and expensive due to the requirement of experienced scientists. With the advancement in chromosomal microarray analysis, some argue that it can replace the conventional cytogenetic testing. The main advantage of using array-based technologies is that it analyzes the total DNA from all the cells in the sample. There have been several studies reporting different levels of mosaicism that the array-based technologies can detect. However, majority of these studies agree that low levels (<10%) may not consistently be detected by array-based technologies. A handful of cases with 10 to 20% mosaicism was reported to be only detected by aCGH and not by conventional cytogenetic analysis. 8 Furthermore, a scarce number of studies reported low level mosaicism of whole chromosomes, such as detection of 5% trisomy 8 mosaicism only by chromosomal microarray analysis. 8 aCGH detection of mosaic cases of terminal and interstitial deletions (<2Mb) have been reported previously. 15 However, since karyotyping analysis failed to show these deletions, the percentage of mosaicism is not known. 15 Here, we report the genetic diagnosis of a 16-year-old apparent female. The cytogenetic analysis of this patient was in concordance with variant Turner syndrome of 52% isochromosome Xq, 22% terminal deletion of Xq11.2 region, and 15% monosomy X. However, aCGH results did not show any mosaicism, rather a 57,252kb deletion on Xp22.33 was detected suggesting the deletion of the *SHOX* gene. The deletion of this gene was also confirmed by fluorescence in situ hybridization analysis. Therefore, the use of aCGH analysis was shown to be more advantageous in detecting the small deletions including detection of gene deletions that may have an effect on the phenotype of the patients. Although previous studies suggest that as low as 10% mosaicism can be detected by aCGH, this case has shown that partial monosomies may not be successfully detected. Even though aCGH has been widely used in detecting both structural and numerical chromosomal abnormalities, its inability in detecting mosaicism impedes its uses. In the absence of routine cytogenetic investigation, the mosaicism would have been missed in this patient. Therefore, the scientists have to keep in mind that aCGH may miss the correct rates of mosaicism, especially at low levels of partial chromosomes. Correct genetic diagnoses play a crucial role since it both affect the patients' own life and the parents. In this case, if the parents of this patient consider future pregnancies, genetic counseling should be offered to the couple not only due to the mosaicism observed in the patient but also due to the inversion detected in the male partner. In case, the couple considers further reproduction, they then can choose from different reproductive options, such as preimplantation genetic diagnosis to select a euploid embryo or prenatal diagnosis.

In conclusion, although aCGH has become the preferred method in detecting numerical abnormalities and unbalanced chromosomal rearrangements, its use is hindered by its failure of detecting mosaicism, especially low-level mosaicism. Therefore, as shown with this patient as well, although the resolution of the aCGH is higher and advantageous in detecting gene deletions, the cytogenetic investigation is still the first in line to detect mosaicism.
